# Lactobacillus Eats Amyloid Plaque and Post-Biotically Attenuates Senescence Due to Repeat Expansion Disorder and Alzheimer’s Disease

**DOI:** 10.3390/antiox13101225

**Published:** 2024-10-12

**Authors:** Suresh C. Tyagi

**Affiliations:** Department of Physiology, University of Louisville School of Medicine, Louisville, KY 40202, USA; suresh.tyagi@louisville.edu

**Keywords:** folate 1-carbon metabolism, CAA, ATP-citrate lyase, gene writer, eraser, RNA editor, Piezo

## Abstract

Patients with Alzheimer’s disease and related dementia (ADRD) are faced with a formidable challenge of focal amyloid deposits and cerebral amyloid angiopathy (CAA). The treatment of amyloid deposits in ADRD by targeting only oxidative stress, inflammation and hyperlipidemia has not yielded significant positive clinical outcomes. The chronic high-fat diet (HFD), or gut dysbiosis, is one of the major contributors of ADRD in part by disrupted transport, epigenetic DNMT1 and the folate 1-carbon metabolism (FOCM) cycle, i.e., rhythmic methylation/de-methylation on DNA, an active part of epigenetic memory during genes turning off and on by the gene writer (DNMT1) and eraser (TET2/FTO) and the transsulfuration pathway by mitochondrial 3-mercaptopyruvate sulfur transferase (3MST)-producing H_2_S. The repeat CAG expansion and m^6^A disorder causes senescence and AD. We aim to target the paradigm-shift pathway of the gut–brain microbiome axis that selectively inhibits amyloid deposits and increases mitochondrial transsulfuration and H_2_S. We have observed an increase in DNMT1 and decreased FTO levels in the cortex of the brain of AD mice. Interestingly, we also observed that probiotic lactobacillus-producing post-biotic folate and lactone/ketone effectively prevented FOCM-associated gut dysbiosis and amyloid deposits. The s-adenosine-methionine (SAM) transporter (SLC25A) was increased by hyperhomocysteinemia (HHcy). Thus, we hypothesize that chronic gut dysbiosis induces SLC25A, the gene writer, and HHcy, and decreases the gene eraser, leading to a decrease in SLC7A and mitochondrial transsulfuration H_2_S production and bioenergetics. Lactobacillus engulfs lipids/cholesterol and a tri-directional post-biotic, folic acid (an antioxidant and inhibitor of beta amyloid deposits; reduces Hcy levels), and the lactate ketone body (fuel for mitochondria) producer increases SLC7A and H_2_S (an antioxidant, potent vasodilator and neurotransmitter gas) production and inhibits amyloid deposits. Therefore, it is important to discuss whether lactobacillus downregulates SLC25A and DNMT1 and upregulates TET2/FTO, inhibiting β-amyloid deposits by lowering homocysteine. It is also important to discuss whether lactobacillus upregulates SLC7A and inhibits β-amyloid deposits by increasing the mitochondrial transsulfuration of H_2_S production.

## 1. Introduction

The significance of this review provides a compelling and convincing case that dysbiosis and dysregulation of homocysteine (Hcy) metabolism represents the dominant mechanism whereby dysbiosis leads to detrimental changes in the brain cortex’s pial vessels and metabolism. Recent research shows that the imbalance between good vs. bad microbial population, especially in the gut, causes systemic diseases. Thus, an appropriate balance of the gut microbiota (eubiosis over dysbiosis) needs to be maintained for normal health [1]. However, diseases such as metabolic syndrome, inflammatory bowel disease, diabetes, obesity, and hypertension in the dysbiotic gut environment tend to prevail [1].

**Figure 1:**
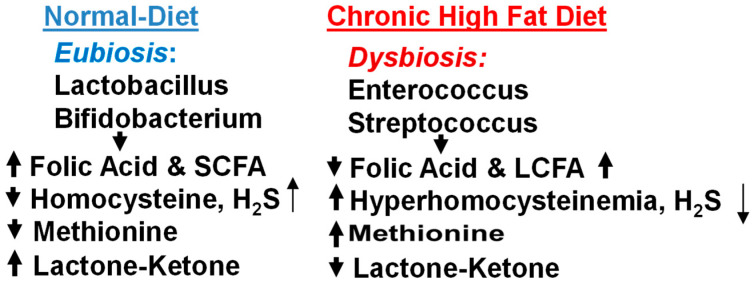
Chronic high fat dysbiosis diet leads to increase methionine and long-chain fatty acids (LCFA). This causes hyperhomocysteinemia (HHcy), lowers short chain fatty acids (SCFA), folate, ketone/lactone, hydrogen sulfide (H_2_S). The probiotic lactobacillus reveres.

The high-fat dysbiosis diet (HFD) leads to cerebral vascular and heart diseases. Interestingly, according to the homocysteine theory, there is one diet high in animal protein and low in B vitamins—which occur in many foods but are very easily destroyed by processing—a diet of meat, cheese, milk, white flour, and foods that are canned, boxed, refined, processed, or preserved. This suggests a strong connection between diet and cerebrovascular and heart disease, but one that is a different path from cholesterol. The homocysteine theory considers atherosclerosis a disease of what McCully calls protein intoxication [2,3]. The cholesterol theory (sometimes called the lipid theory) instead demonizes fats. Since proteins and fats often occur in the same foods, the potential dietary treatments for high homocysteine and high cholesterol levels are similar, with the following distinction: the anti-homocysteine diet focuses on what should be eaten, as a preventive, while the anti-cholesterol diet focuses on what should be avoided, as a precipitator. Thus, a diet with lower homocysteine levels would include many natural sources of B vitamins like fresh fruits and vegetables and would limit animal protein. The cholesterol-reducing diet would limit foods high in saturated fats and cholesterol, like eggs, meat, and butter (Figure 1). Unfortunately, the latter is more commercially popular.

**Is brain amyloidosis by proteinopathies more detrimental than atherosclerosis**? Atherosclerosis is primarily cholesterol engulfed by macrophage/foam cells along with lipid/ApoE in core atheroma held with a thin fibrosis cap, which is prone to rupture by MMPs. In addition, SMCs can induce apoptosis, and the colonial SMC causes thickening of media, and hypertrophy and fibrosis. In **amyloidosis**, an accumulation of proteins occurs. **Proteinopathies** include beta-amyloids, tau, TDP-43, alpha-synuclein, and lipids building plaque. Interestingly, both cause a chronic decrease in blood flow; however, in the brain, this decrease in blood flow causes VCID, leading to SMC apoptosis, whereas in other organs, this is compensated until the plaque is ruptured and then acute thrombosis with no flow occurs. Interestingly, the transactive DNA-binding protein 43 (TDP-43)’s immunoreactivity is associated with sporadic Alzheimer’s disease (AD) and Down’s syndrome (DS) [4,5]; here, the mechanisms are unclear. Interestingly, a growth arrest in DNA damage protein 45 (GADD45) is associated with MMP-13 [6].

The downstream micro-vessels are more responsive to flow-mediated vaso-elastic compliance. Therefore, atherosclerosis in a conduit artery is not that detrimental; it is the decrease in flow to downstream small micro-vessels that causes endothelial dysfunction and VCID. **Homocysteine (Hcy, a consequence of proteinopathies)** has a direct damaging role in the endothelium as compared with cholesterol or lipids, suggesting that Hcy contributes significantly to **proteinopathy/arteriosclerosis** and VCID. Interestingly, the conversion of this toxic Hcy to H_2_S is beneficial to downstream micro-vessel endothelial vaso-elastic compliance and H_2_S dissolution of the amyloid plaques, as this project elutes.

Alzheimer’s disease (AD/ADRD) is multifactorial in the sense that it is associated with amyloid beta [7] and tau [8] depositions. Interestingly, folic acid attenuates beta amyloid deposits [9,10,11]. The interest in molecular clean-up mechanisms to remove plaque in Alzheimer’s disease is suggested [12]. A study also suggested that eubiotic bacteria degrades cholesterol [13] and may reduce plaque. Here, we propose to target the paradigm-shift pathway in the gut–brain microbiome axis in which a eubiotic bacteria lactobacillus treatment selectively inhibits amyloid deposits in part by producing folate (an epigenetic regulator) and H_2_S (improves mitochondrial bioenergetics) and selectively inhibits amyloid deposits in Alzheimer disease. **Additionally, folic acid and H_2_S inhibit the beta-amyloid plaque accumulation** [9,10,11].

The increased levels of proprotein convertase/secretase (BACE1) [14,15,16] are associated with AD. We have demonstrated the role of proteinase/convertase and anti-proteinase in the accumulation of homocysteine (Hcy, i.e., hyperhomocysteinemia (HHcy)) by the disrupted folate-1 carbon metabolism (FOCM) cycle; in vascular stiffness [15], contributing to the enlarged perivascular space (EPVS) [17,18,19,20]; and in vascular contributions to cognitive impairment and dementia (VCID) via the activation of matrix metalloproteinase-9 (MMP-9) [21,22,23]. Using MMP-9 knockout (MMP-9KO) mice, we observed attenuation in HHcy-induced BBB leakage and EPVS [24,25].

HHcy is associated with VCID, AD and ADRD in a dose-dependent (causative) manner [26,27,28,29,30,31,32,33,34,35,36,37,38]. Further, HHcy induces seizures in animals [37] and is associated with vascular dementia and AD in humans [30,38].

Children born with severe HHcy have intellectual disabilities and do not live past teenage years. However, children with mild/moderate HHcy live relatively asymptomatic with mild intellectual disabilities. That is why, to reduce HHcy, folic acid, a probiotic, is prescribed during pregnancy to avoid detrimental effects of HHcy and to mitigate intellectual disabilities and prevent neural tube defects during embryonic development [39,40,41].

The Hcy plasma levels are higher in males than premenopausal females; however, they become similar in post-menopausal females [28]. Interestingly, gut dysbiosis is associated with the degradation of estrogen and depression in premenopausal women [42].

**Figure 2:**
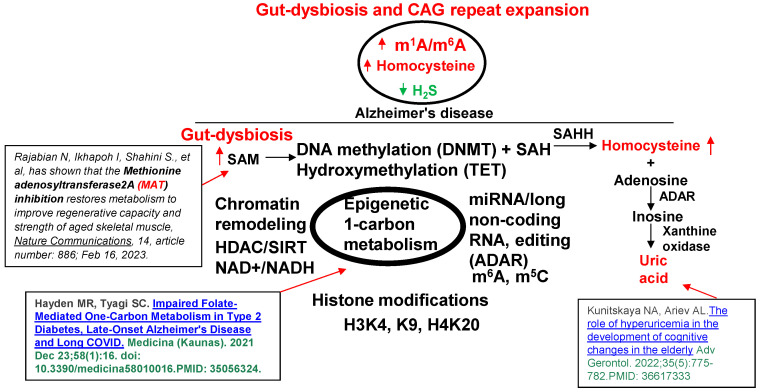
Schematics of how gut dysbiosis leads to epigenetic methylation alterations and causes Alzheimer’s disease (AD). ADAR, adenosine deaminase acting on RNA; CAG, cytidine-adenosine-guanidine), m^1^A, methyl-1-adinosine; SAM, s-adenosine methionine; SAH, s-adenosine homocysteine; SAHH, s-adenosine homocysteine hydrolase; DNMT, DNA methyltransferase; TET, ten eleven translocators; HDAC, histone de-acetylase; SIRT, Histone-protein de-acetylase; H3K4, histone-3 lysine 4 [21,60,61].

**Figure 3:**
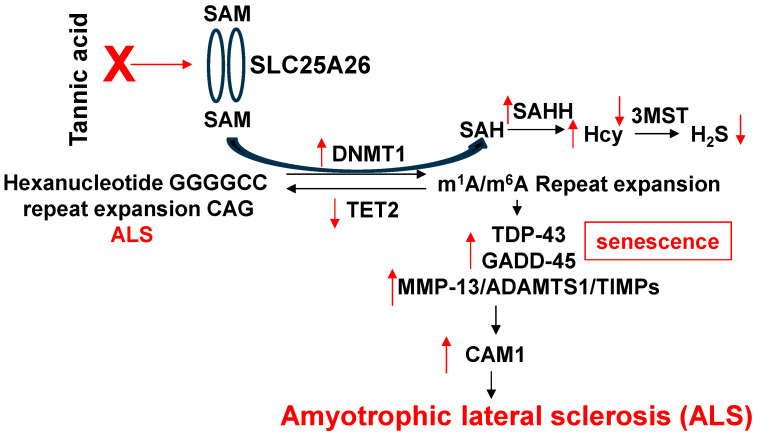
Repeat nucleotide sequences (CAG) cause random mutations, leading to ALS and AD. The tannic acid inhibits transporter SLC25A and mitigates ALS and AD.

In addition, telomere shortening via epigenetic methylation [43,44] by the gene writer (DNMT1) and hydroxylation/de-methylation by the gene eraser (TET2) is also linked to AD (Figure 2 and Figure 3). A direct relationship exists between a healthy lifestyle and eubiosis. However, during dysbiosis, there is an increase in Hcy levels associated with cerebrovascular dementia (CVD) and AD [45,46,47,48]. Figure 2 **elicits how this project fills the gaps in AD research**. A chronic high-methionine diet, a substrate for homocysteine (Hcy), contributes to AD and ADRD [49,50,51,52]. The s-adenosine-methionine (SAM) transporter (SLC25A) was increased by hyperhomocysteinemia (HHcy) [53,54,55] and cysteine was transported by SLC7A5 [56]. Interestingly, we and others have shown that the conversion of methionine to Hcy is regulated by the epigenetic folate 1-carbon metabolic (FOCM) pathway (Figure 3) [57,58,59]. The inhibition of methionine adenosyltransferase2A (MAT2A) restores metabolism to improve regenerative capacity and strength in aging muscles [60].

**Figure 4:**
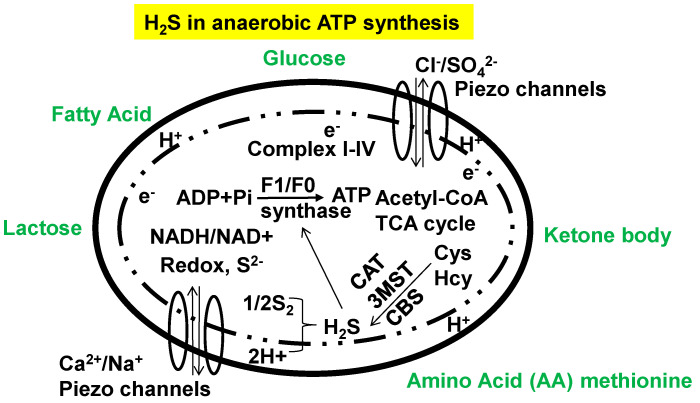
During ischemic conditions such as COPD, sleep apnea and decrease pulmonary function, initially mitochondrial synthesizes H_2_S and coups with dys-bioenergetics. COPD, chronic obstructive pulmonary diseases; TCA, tri-carboxylic acid; CAT, cysteine transferase; 3MST, 3mercaprtopyruvate sulfotransferase; CBS, cystathionine beta transferase; Piezo, mechano-thermal Na/Ca/Mg and transient receptor potential receptor/channels.

Recent studies show that intellectual disabilities are caused by the hypermethylation of genes [62,63], which generates Hcy [59]. There is rhythmic methylation/de-methylation during the mitochondrial TCA cycle by the epigenetic gene writer (DNMT) and erasers (TET and FTO) [16] (Figure 4).

Although epidemiological studies have also indicated that HHcy is a contributing factor for the development of atherosclerotic lesions and hypertension [64,65], in fact, HHcy synergizes with an increase in blood pressure and induces endothelial dysfunction by decreasing the bioavailability of endothelial NO [66,67,68]. Although we and others demonstrated cognitive impairment and cerebral vascular dementia/leakage in CBS-/+-HHcy and AD mice [69,70,71,72], the connection between active gene writers and erasers and an increase in the formation of Hcy is unclear. In addition, HHcy instigates thromboembolism and cerebral vascular diseases [33,34]. Reductions in Hcy levels are associated with reduced carotid artery restenosis events after angioplasty [35,36].

**Figure 5:**
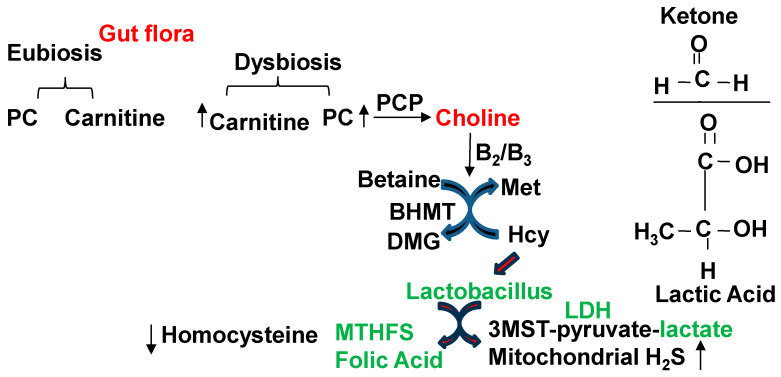
The probiotics lactobacillus mitigates folate deficiency and improves mitochondrial pyruvates and H_2_S levels, post-biotically. PCP, phosphatidylcartinine phosphatase; BHMT, betaine homocysteine methyl transferase.

Epigenetic DNA methylation via FOCM and choline (Figure 5) [58] are a part of epigenetic memory, i.e., gene imprinting and off-printing during embryogenesis, development, and AD [21]. This epigenetic memory is retained in a transgenerational manner [73]. Treatment with tri-directional *Lactobacillus rhamnosus* (i.e., folate- and lactate-producing post-biotics) reverses the dysbiosis-induced cerebral vascular injury in part by increasing mitochondrial sulfur transsulfuration (CBS, CSE, 3MST, LDH, H_2_S, i.e., mitochondrial biogenesis) [74]. The H_2_S again protects against AD [75]. Therefore, it is important to identify microbiome-derived post-biotic metabolites such as folate and ketone bodies for their beneficial effects (Figure 5) [76,77,78,79,80,81,82,83,84].

## 2. Conclusions and Future Directions

Dysbiosis disrupts the gene writer/eraser ratio [85,86,87,88] and shifts the equilibrium towards vascular dementia and cognitive impairment. The hypothesis that gut dysbiosis in AD induces epigenetic gene writers DNMT and SAHH and decreases erasers TET and FTO, creating HHcy, is novel. HHcy decreases mitochondrial sulfur metabolism, i.e., transsulfuration by CBS, CSE and 3MST, causing oxidative stress (decrease in H_2_S) and MMP activation. Lactobacillus, a probiotic that produces folic acid, mitigates dysbiotic 1-carbon metabolism by re-methylation of Hcy to methionine, HHcy and vascular dementia. The metabolites of probiotic lactobacillus, such as lactones, are also beneficial as fuel for mitochondria. Lactobacillus is a novel therapy based on its safety, is inexpensive and is a noninvasive way that can mitigate ADRD. Organoids such as vascular mapping of the brain [89,90,91] and our X-ray imaging of micro-vessels in the brain clearly demonstrate that micro-vessels in brains with AD are narrower than the WT controls. This may be due in part to the fact that mitochondrial oxidative/reductive imbalances are due to dysfunctional mitochondrial sulfur metabolism [92] and transsulfuration. The tri-directional lactobacillus (i.e., folic acid and lactate/lactone/ketone body producers) attenuates HHcy and amyloid deposits [9,10,11] and improves mitochondrial function. This mitigates microvascular leakage, VCID and AD [93,94,95,96,97,98,99,100,101].

**Figure 6:**
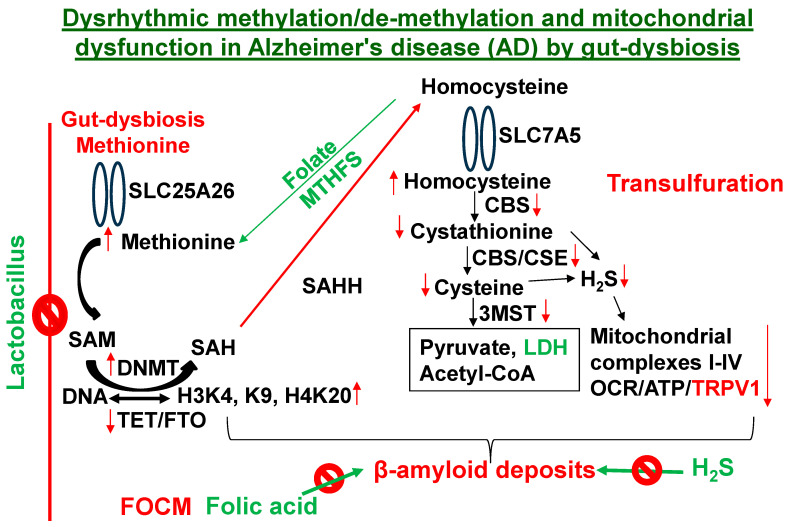
The hypothesis is that the chronic gut-dysbiosis induces SLC25A, **gene writer (DNMT1)**, HHcy and decreases gene eraser (TET2/FTO), leading to decrease SLC7A and mitochondrial transsulfuration H_2_S production and bioenergetics. Lactobacillus, a tri-directional, **folic acid (an inhibitor of beta amyloid deposits**, reduces Hcy levels), and lactate ketone-body (fuel for mitochondria) producer **increases SLC7A and** H_2_S production and inhibits amyloid deposits.

**It is important to consider the inhibition of senescence and β-amyloid deposits via tri-directional probiotic strategies for the attenuation of repeat expansion disorder in Alzheimer’s disease.** The repeat expansion disorder (RED) in the CAG codon and methylation of m^1^A and m^6^A in this codon contribute to defective DNA repair, senescence, and dementia [102,103,104,105,106,107,108]. The mechanism is unclear. The hypothesis is that, in AD, the methylation of RED increases Hcy and TDP43/GADD45/MMP/ADAMTS, causing senescence and decreasing H_2_S [85,86,87]. This causes CAM1/β-amyloid deposits, VCID and ADRD. The probiotic (PB), lactobacillus, produces post-biotic folate and increases H_2_S levels, and inhibits senescence, β-amyloid deposits and VCID/ADRD (Figure 6). It is important to determine whether lactobacillus inhibits senescence by decreasing DNMT1, m^1^A, m^6^A, TDP-43, GADD45, MMP-13, and ADAMTS1, and increasing TET2, 3MST, H_2_S and TIMPs in RED and Alzheimer’s disease. Also, it is novel to determine whether lactobacillus inhibits β-amyloid deposits and CAM1 by increasing folate and H_2_S for the attenuation of RED, VCID and Alzheimer’s disease.

This review presents an innovative hypothesis to test the role of repeat expansion disorder in the development of senescence, dementia, and AD through methylation and HHcy. This is a consequence of the disruption of “1-carbon metabolism” in RED via the modulation of epigenetics (hypermethylation and HHcy) [102,103,104,105,106,107,108]. In addition, the therapeutic effect of probiotic treatment will be tested to reverse these effects. The elucidation of the mechanisms through which RED and hypermethylation affect brain senescence, memory and health is important, and positive findings will support the relevance and impact of RED and hypermethylation in brain disorders. Although the use of probiotics as a therapeutic alternative for AD has been previously studied in pre-clinical models and clinical trials, there is an unmet need for a mechanism-based, simple, and safe therapy. Here, we suggest that a probiotic that produces folate (lowers Hcy and inhibits beta amyloid deposits) and lactate (a ketone body, fuel for mitochondria, and increases H_2_S) can potentially mitigate the consequences of RED, Alzheimer’s disease and related dementia.

## Abbreviations

AD, Alzheimer’s disease; ADRD, Alzheimer’s disease and related dementia; ADAMTS, a disintegrin and metalloproteinase thrombospondin domain 1; CAA, cerebral arterial angiopathy; CAM1, cell adhesive molecule 1; DNMT1, DNA methyl transferase 1; 3MST, 3-mercaptopyruvate sulfur transferase; RED, repeat expansion disorders; m^1^A, 1-methyl adenosine; m^6^A, 6-methyl adenosine; CAG, cytosine adenosine guanidine; MMP, matrix metalloproteinase; Hcy, homocysteine; H_2_S, hydrogen sulfide; TDP43, transactive DNA protein-43; GADD-45, growth arrest DNA damage protein-45; SMC, smooth muscle cell; TET2, ten eleven translocator 2; TIMPs, tissue inhibitors of metalloproteinases; VCID, vascular contributions to cognitive impairment and dementia.
